# Interventricular Septum Diverticulum: A Multimodality Imaging Approach to Diagnosis

**DOI:** 10.3390/diagnostics15212814

**Published:** 2025-11-06

**Authors:** Romain Van der Linden, Mohamed El Mallouli, Chirine Liu, Maxime Goldfinger, Georgiana Pintea Bentea

**Affiliations:** 1 Department of Cardiology, CHU UCL Namur Sainte Elisabeth, 5000 Namur, Belgium; romain.vdlv@gmail.com (R.V.d.L.); mohamed.el.mallouli@ulb.be (M.E.M.); chirine.liu@gmail.com (C.L.); 2 Department of Radiology, CHU Brugmann, 1020 Brussels, Belgium; maxime.goldfinger@chu-brugmann.be

**Keywords:** ventricular outpouching, interventricular septum diverticulum, multimodality imaging

## Abstract

We report the case of a 57-year-old female smoker who presented to the cardiology department with atypical chest pain. The transthoracic echocardiography revealed an interventricular septal diverticulum. To further characterize this finding, additional investigations were performed, including coronary CT angiography, cardiac magnetic resonance imaging, and coronary angiography. These examinations excluded the presence of an aneurysm, pseudoaneurysm, or ventricular septal defect, and confirmed the diagnosis of a congenital interventricular septal diverticulum. Following multidisciplinary cardiology and surgical team discussion, a conservative management approach was adopted. Left ventricle outpouching are rare cardiac malformations. Differentiating between diverticulum, aneurysm, and pseudoaneurysm using multimodal imaging is crucial for clinical follow-up and prognosis.

**Figure 1:**
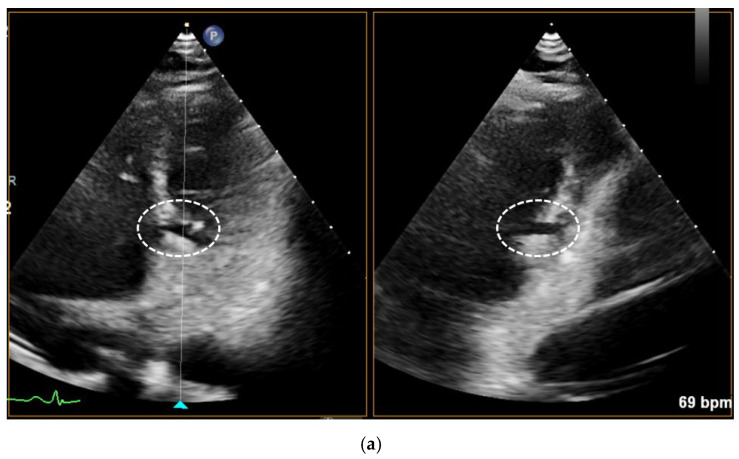

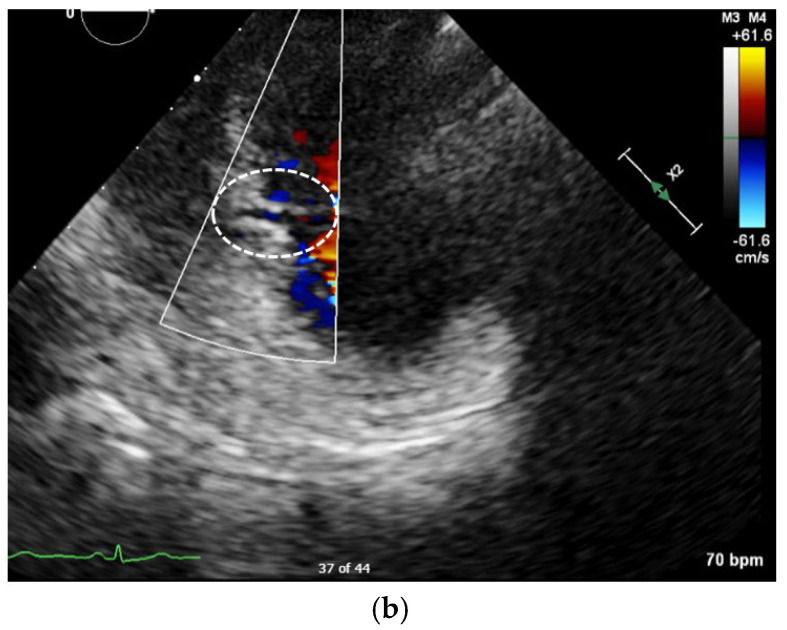
We describe the case of a 57-year-old female smoker who presented in cardiology consultation with atypical chest pain over the last few weeks. The EKG proved to be normal; however, the transthoracic echocardiography performed during the consultation revealed an interventricular septum diverticulum in the pars muscularis of the inferoseptal wall, as illustrated by the circled areas in the modified four chamber views in Panel (**a**). There were no signs of ventricular septal defect: the color Doppler showed no visible flow passing perpendicularly to the interventricular septum through the diverticulum, as shown by the circled area in Panel (**b**), the left ventricle was not dilated, the patient was hemodynamically stable. The echocardiography incidences were modified in order to better illustrate the pathology in question, as such not being the standard incidences. Furthermore, the patient did not exhibit a heart murmur suggestive of this pathology during the clinical examination. In addition, the blood work-up showed normal levels of troponin and NTpro BNP.

**Figure 2:**
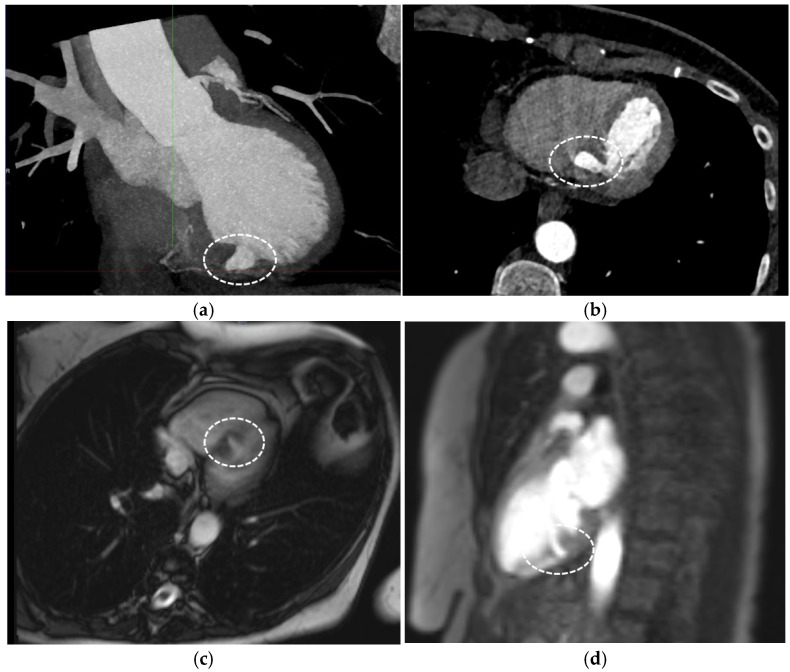

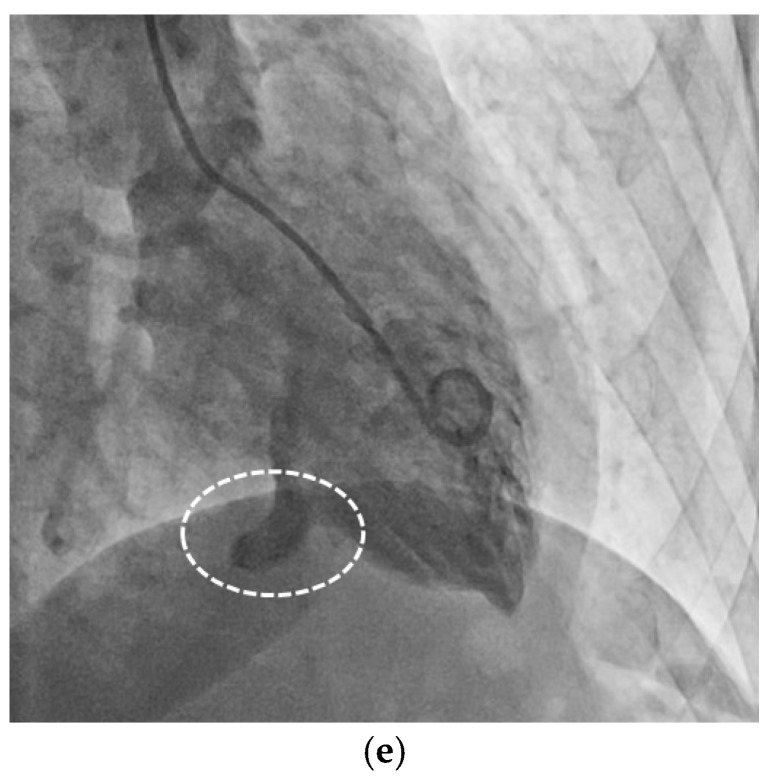
Given the discordant clinical, biological, and echocardiography findings, in order to further characterize the interventricular septum diverticulum and its clinical significance, additional investigations were performed [1], including a coronary CT angiography that showed normal coronary arteries and the interventricular septum outpouching (circled areas in Panels (**a**,**b**)), however, not being able to distinguish between an aneurysm or a pseudoaneurysm. Hence, we carried out a cardiac magnetic resonance, which showed a normal biventricular contractility and the presence of a muscular layer lining the outpouching (circled areas in Panels (**c**,**d**)), as such excluding the hypothesis of a pseudoaneurysm. Moreover, the cardiac magnetic resonance revealed that the septal outpouching was bordered by healthy myocardium, with no evidence of fibrosis, and was in itself contractile without a paradoxical movement, hence refuting the diagnosis of aneurysm [2]. A coronary angiography was performed, confirming a normal coronary artery network and no ventricular septal defect (circled area in Panel (**e**)). The multimodality imaging performed clarified the diagnosis of an interventricular septum diverticulum in this patient. The main objective of this paper was to illustrate the importance of multimodality imaging in characterizing the structure that we observed at the level of the interventricular septum: either diverticulum, aneurysm, pseudoaneurysm, or septal ventricular defect, as their pathological significance and management are very different. The echocardiography in itself, although successful in identifying an abnormality, was insufficient to characterize the exact pathological entity. The cardiac magnetic resonance helped us to exclude the hypothesis of a pseudoaneurysm. Coronary angiography allowed us to assess with accuracy the contractility of the interventricular formation, the absence of septal ventricular defect, and the shape of the inlet, as such contributing in determining the final diagnosis of septal interventricular diverticulum. In addition, it offered certain information regarding the epicardial status of the coronary artery disease. It is worth mentioning that there are no comparative studies in the literature between the different imaging modalities to assess a left ventricle diverticulum, especially situated in the interventricular septum. Left ventricle diverticulum is a rare type of cardiac malformation, found in previous retrospective analysis of reported prevalence as low as 0.04% [3]. Furthermore, the localization at the level of the interventricular septum is one of the rarer occurrences, found in 2.5% of cases with left ventricular diverticulum [2]. Furthermore, it is often associated with other types of cardiac malformations. We reexamined the performed investigations that confirmed the absence of associated cardiac malformations. In consequence, we retained the diagnosis of isolated congenital interventricular septum diverticulum. This condition is usually asymptomatic, however, can be seldom proarrhythmogenic [3]. During the follow-up consultation, the patient had a complete resolution of her chest pain, suggesting that it was not linked to the diverticulum. Even in the absence of palpitation, we performed a Holter rhythm monitoring to detect potential arrhythmias originating from the diverticulum, and there were none. Following multidisciplinary cardiology and surgical team discussion, a conservative management approach was adopted. Previous findings indicate that the prognosis of left ventricular aneurysm is significantly worse than that of left ventricular diverticulum in terms of cardiac death and ventricular tachycardia/fibrillation [2]. The cornerstone of the management of this patient was relying on the multimodality imaging in order to differentiate between diverticulum, aneurysm, pseudoaneurysm, and septal ventricular defect, which was crucial for the clinical management, follow-up, and prognosis [4,5].

## Data Availability

The data presented in this study are available on request from the corresponding author due to privacy reasons.

## References

[1] Cresti A., Cannarile P. (2018). Multimodality Imaging and Clinical Significance of Congenital Ventricular Outpouchings: Recesses, Diverticula, Aneurysms, Clefts, and Crypts. J. Cardiovasc. Echogr..

[2] Ohlow M.A., von Korn H. (2015). Characteristics and outcome of congenital left ventricular aneurysm and diverticulum: Analysis of 809 cases published since 1816. Int. J. Cardiol..

[3] Mayer K., Candinas R. (1999). Congenital left ventricular aneurysms and diverticula: Clinical findings, diagnosis and course. Schweiz. Med. Wochenschr..

[4] Ohlow M.A., Lauer B. (2012). Long-term prognosis of adult patients with isolated congenital left ventricular aneurysm or diverticulum and abnormal electrocardiogram patterns. Circ. J..

[5] Marijon E., Ou P. (2006). Diagnosis and outcome in congenital ventricular diverticulum and aneurysm. J. Thorac. Cardiovasc. Surg..

